# Pharmacist-Initiated Team-Based Intervention for Optimizing Guideline-Directed Lipid Therapy of Hospitalized Patients With Acute Coronary Syndrome: Pilot Study Using a Stepped-Wedge Cluster Design

**DOI:** 10.2196/58837

**Published:** 2025-03-28

**Authors:** Gayle L Flo, Mateo Alzate Aguirre, Benjamin R Gochanour, Kristin J Hynes, Christopher G Scott, Angela L Fink, Adelaide M Arruda-Olson

**Affiliations:** 1 Department of Cardiovascular Medicine, Mayo Clinic, 200 First Street Southwest, Rochester, MN, 55905, United States, 1 507-284-2511; 2 Ascension St. John Hospital, Detroit, MI, United States; 3 Department of Cardiovascular Medicine, Mayo Clinic, Rochester, MN, United States; 4 Department of Quantitative Health Sciences, Mayo Clinic, Rochester, MN, United States; 5 Pharmacy Services, Mayo Clinic, Rochester, MN, United States; 6 Department of Nursing, Mayo Clinic, Rochester, MN, United States

**Keywords:** coronary disease, follow-up studies, lipids, myocardial infarction, statins

## Abstract

**Background:**

Clinical guidelines recommend high-intensity statin therapy for patients with acute coronary syndrome (ACS). However, high-intensity statins have been underused in this population.

**Objective:**

The objective of this study was to evaluate the feasibility of a pharmacist-initiated, team-based intervention for the delivery of individualized, guideline-directed, lipid-lowering therapy for patients with ACS.

**Methods:**

Patients admitted with ACS to cardiology hospital services at Mayo Clinic from August 1, 2021, to June 19, 2022, were assigned to a pharmacist-initiated, team-based intervention group or control group using a stepped wedge cluster study design. For the intervention group, pharmacists reviewed electronic health records and provided recommendations for lipid lowering therapy in hospital and at follow-up. In the control group, patients received usual care. Neither care team, nor study team were blinded to study assignments. The primary outcome was the proportion of patients with ACS discharged on high-intensity statins in the intervention group compared to controls. Secondary outcomes were (1) proportion of patients in the intervention group with a specific templated pharmacist intervention note in their electronic health records, (2) frequency of low-density lipoprotein (LDL) measurements in hospital, (3) proportion of patients with information related to lipid follow-up in their discharge summary, and (4) proportion of patients that received LDL monitoring at the outpatient follow-up 4 to 12 weeks post discharge.

**Results:**

There were 410 patients included in this study (median age 68, IQR 60-78 years) of whom 285 (69.5%) were male. Of the 402 patients alive at discharge, 355 (88.3%) were discharged taking a high-intensity statin, with no significant difference (*P*=.89) observed between groups. Lipid levels were measured in the hospital for 176/210 (83.8%) patients in the intervention group and 155/200 (77.5%) patients in the control group (*P*=.14). Fifty-four of 205 (26.3%) intervention patients alive at discharge had lipid-related recommendations in their discharge summary compared to 27/197 (13.7%) controls (*P*=.002). Forty-seven of 81 (58%) patients with lipid management recommendations provided in the discharge summary had LDL measured in the follow-up period compared with only 119/321 (37.1%) patients without these recommendations (*P*=.001). Of the 402 patients who survived to discharge, 166 (41.3%) had LDL measured at follow-up; the median LDL level was 63.5 (IQR 49-79) mg/dL, and distributions were similar by group (*P*=.95). Only 101/166 (60.8%) patients had follow-up LDL values below the target of 70 mg/dL.

**Conclusions:**

During hospitalization, there was no group difference in the primary outcome of high-intensity statin therapy. Feasibility of an effective pharmacist-initiated intervention for improvement of lipid management was demonstrated by entry of recommendations in the discharge summary and related adjustment in outpatient statin therapy. The main opportunity for future improvement in lipid management of patients with ACS is in longitudinal patient follow-up.

## Introduction

Acute coronary syndrome (ACS) includes non–ST-segment elevation myocardial infarction, ST-segment elevation myocardial infarction, and unstable angina [1-3]. Current estimates show approximately 605,000 new and 200,000 recurrent infarctions each year in the United States [4]. In 2020, there were 577,275 hospital discharges for ACS diagnosis [4]. Data from a Swedish registry revealed that approximately 20% of 97,254 patients who survived a myocardial infarction experienced another ischemic cardiac event within 24 months [5]. The 5-year mortality for ACS from large United Kingdom and Belgian studies ranged from 19% to 22% [6,7].

High-intensity statin therapy in the setting of ACS yields significant mortality benefit [8,9]. Hence, clinical practice guidelines recommend statin therapy for all patients with ACS [10,11]. In addition to decreasing low-density lipoprotein (LDL) levels, statins also promote improvement of endothelial function, decrease of platelet aggregation, and reduction of vascular inflammation [12]. LDL levels are used to monitor the intensity of therapy [13-15]. Guideline-directed therapies, including statins have been underused by patients with ACS [16]. For example, in a large cohort of 690,524 patients with recent ACS, less than half were on any statin therapy, and of those, only 20% were on high-intensity statins [17]. Another study which included 7802 patients with ACS showed that only one-third were prescribed a high-intensity statin at index hospitalization, and of those, only half were on such therapy at 1 year of follow-up [18].

Prior studies have demonstrated improved use of guideline-directed medical therapy by using team-based care delivery models. One prior study achieved sustained decreases in LDL levels to a specified target when pharmacists managed therapy for patients with coronary heart disease in the outpatient setting [19]. Another study showed that a pharmacist-initiated, team-based intervention with admission and predischarge medication reconciliation resulted in better adherence to guideline-directed therapy and reduced readmissions for heart failure [20]. The need to develop care delivery models to promote improved achievement of LDL targeted therapy is further supported by the work of Basaran et al [21] who analyzed data from 873 patients with diabetes from the EHPESUS registry which revealed that only 19.5% of the primary prevention and 7.5% of the secondary prevention groups were at LDL goal.

We hypothesize that a team-based inpatient care delivery model with processes that promote use of guideline-directed medical therapy for lipid management may improve outcomes for patients with ACS. An important unmet need exists to optimize lipid-lowering therapy for patients with ACS. Accordingly, the aim of this pilot study was to evaluate the feasibility of a pharmacist-initiated, team-based inpatient intervention for delivery of individualized, guideline-directed, lipid-lowering therapy recommendations for patients with ACS and to collect preliminary data on effectiveness.

## Methods

### Recruitment

This study was performed from August 1, 2021, to June 19, 2022, in 6 cardiology hospital services which admit patients with suspected ACS at Mayo Clinic in Rochester, Minnesota. Patients were included if they had a new diagnosis of ACS, that is, non–ST-segment elevation myocardial infarction, ST-segment elevation myocardial infarction, or unstable angina. Inclusion criteria remained consistent throughout the entire trial.

### Study Design

#### Overview

**Figure 1.**
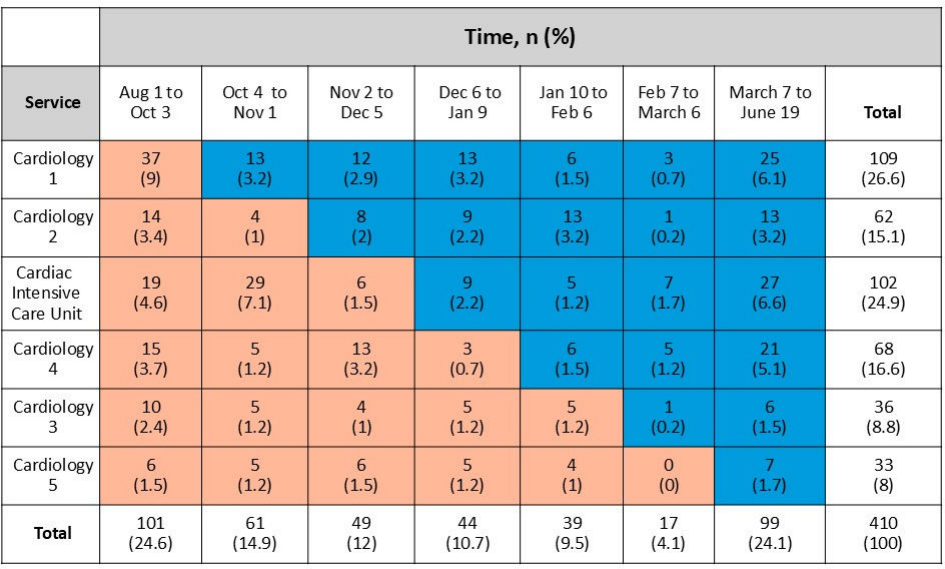
Stepped wedge cluster allocation of patients.

All patients admitted with ACS to cardiology were assigned to the control group (usual care) during the first 2 months of the project. At the beginning of month 3, the cardiology services began crossing over to the intervention group following a stepped wedge design [22] (Figure 1). Hence, each service had exposure to control status and intervention status over this study’s period in longitudinal fashion. Each cluster of patients was unique in that patients with repeat admissions were excluded from this study at subsequent admissions. Neither the care team nor this study’s team were blinded to the intervention status of patients.

Baseline characteristics were collected for all patients enrolled. Data collection occurred via electronic health record (EHR) review after hospital admission with further completion of the datasets throughout this study’s period. Statin therapy was defined as low-intensity (pravastatin, 10 and 20 mg; simvastatin, 10 mg), moderate-intensity (atorvastatin, 10 and 20 mg; pravastatin, 40 and 80 mg; rosuvastatin, 5 and 10 mg; and simvastatin 20‐40 mg), or high-intensity (atorvastatin, 40 and 80 mg; rosuvastatin, 20 and 40 mg). Sample size calculations were not performed. The intent was to collect data for 8 months based on project timeline and resource allocation.

#### Control Group

Patients in the control group received standard care for ACS, which included high-intensity statin therapy as recommended by clinical practice guidelines [10,11]. Each cardiology team was comprised of internal medicine residents and advanced practice providers (nurse practitioners or physician assistants) supervised by cardiologists. These teams collaborated with cardiology pharmacists who provided guidance about lipid therapy. All cardiology hospital pharmacists rotate covering each of the 6 services based on pre-established staffing schedules. The pharmacists were responsible for reviewing the patients’ EHR daily, completing admission and discharge medication reconciliation, and entering recommendations. The pharmacists also rounded with hospital services to collaborate with the team regarding medication management.

#### Pharmacist-Initiated, Team-Based Intervention

The primary objective of the pharmacist-initiated, team-based intervention was to ensure initiation or continuation of high-intensity statins, and the addition of ezetimibe if patients already taking a high-intensity statin had LDL level greater than 70 mg/dL on either most recent outpatient testing or in-hospital testing.

The cardiology pharmacist group consisted of 9 pharmacists who received training and instructions regarding implementation of the intervention in the form of presentations at staff meetings and written documents shared via emails describing project goals and pharmacist roles. At the beginning of each hospital service the cardiologists and team members entering the intervention phase received an email from this study’s team describing the project.

After patients with ACS were admitted to the hospital, the pharmacists reviewed the EHR and interviewed each patient to gather information about adverse effects to statins and evaluate preadmission LDL levels from the EHR. Subsequently, contraindications to statins and adverse effects were documented in the pharmacist EHR note. If a lipid panel was not available from the prior 6 months, the pharmacists recommended checking a lipid panel to the cardiology team. After reviewing lipid levels, the pharmacists provided specific recommendations for the cardiology team members via EHR text messages and verbal communication.

**Figure 2.**
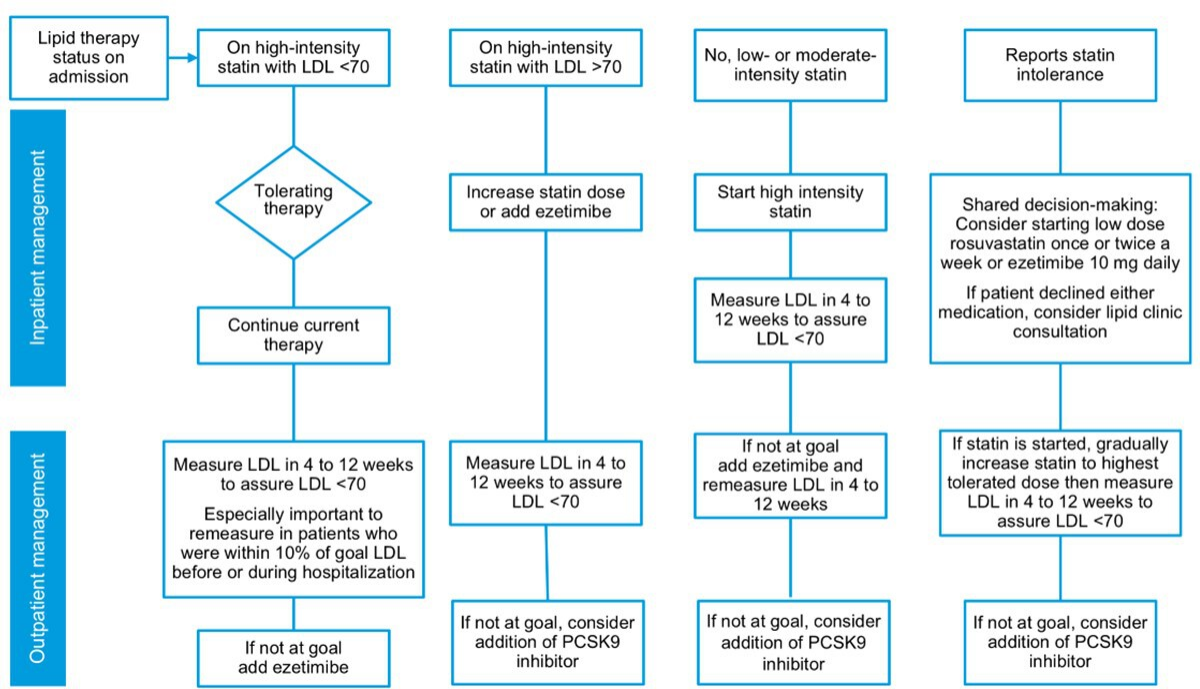
Pharmacist recommendation algorithm. LDL: low-density lipoprotein; PCSK9: proprotein convertase subtilisin/kexin type 9.

The pharmacist recommendation algorithm is summarized in Figure 2. If the patient had an LDL<70 mg/dL and was on high-intensity statin, this medication was continued without change; if the LDL was >70 mg/dL while on a high-intensity statin the options were to increase statin dose or add ezetimibe. If the patient was not on a statin or was taking a moderate-intensity statin therapy, the moderate-intensity statin was discontinued and replaced by a high-intensity statin irrespective of LDL level. If the patient reported prior statin intolerance management options included (1) initiation of low-dose rosuvastatin 5 mg once or twice a week, (2) initiation of ezetimibe only, or (3) patient referral for lipid clinic consultation at the lipid clinic. Each of these processes involved patient-centered shared decision-making for the selection of management strategy.

The pharmacists documented results of their review and recommendations in specially formatted pharmacist intervention notes. These notes recommended lipid testing within 4 to 12 weeks after discharge and treatment modifications if LDL remained greater than 70 mg/dL. Pharmacists requested that cardiology team members include these recommendations in discharge summaries sent to the primary care provider via the EHR. Fidelity with the intervention was evaluated by the presence and content of a templated pharmacist intervention note documented in the EHR.

The pharmacist notes advised repeat lipid measurements at 4 to 12 weeks after hospital discharge, as recommended by the guidelines [10]. However, very few patients underwent testing within 12 weeks. Therefore, the data collection interval was extended to 6 months post hospital discharge. The low frequency of testing by 12 weeks was likely related to clinical decisions and appointment availability in the outpatient clinics. The research team had no influence on scheduling of follow-up appointments.

Follow-up outcomes were obtained by manual review of the EHR within 6 months of hospital discharge. Variables obtained at follow-up were LDL results, test date, and adjustments in lipid therapy made at follow-up. A REDCap (Research Electronic Data Capture, Vanderbilt University 2022) database and Microsoft Excel (Microsoft Corp) were used for data entry and storage.

#### Outcomes

The primary outcome was the proportion of patients with ACS discharged on high-intensity statins in the intervention group compared to the control group. Secondary outcomes were (1) proportion of patients in the intervention group with the specific templated pharmacist intervention note in the EHR, (2) frequency of LDL measurements in the hospital, (3) proportion of patients with information related to lipid follow-up in their discharge summary, and (4) proportion of patients that received LDL monitoring at outpatient follow-up 4 to 12 weeks post discharge.

#### Statistical Methods

Baseline demographic characteristics of the patients were summarized as median (IQR) for continuous and count (proportion expressed as percentage) for categorical variables. Baseline comparisons of continuous variables between groups were made with the Wilcoxon rank-sum test, and comparisons of categorical variables were made with the chi-square or Fisher exact tests.

Preadmission and in-hospital LDL levels were compared by a paired *t* test (2-tailed). The χ^2^ test was used to assess impact of the intervention on the number of patients who had lipid levels measured during hospitalization and the percentage of patients discharged on high-intensity statin therapy. The effect of the intervention on changes made in lipid-lowering therapy from admission to discharge was assessed using a Cochran-Mantel-Haenszel test. Overall rates of admission without lipid therapy compared to discharge without lipid therapy were evaluated by the McNemar test. LDL levels at follow-up were compared by group with the unpaired *t* test. Other follow-up outcome comparisons were made using the χ^2^ test.

A stepped wedge cluster design [22] was used for subject allocation, with the cardiology services as clusters (Figure 1). We evaluated the effects of admission period and cardiology service (rows and columns of Figure 1, respectively) on the outcome variables of interest and found that the results are not likely confounded by these factors. This evaluation was initially performed visually. Subsequently variables were added as covariates in the regression models. No significance or discernable patterns were found; therefore, only the simplified (unadjusted) results are presented herein. For continuous variables, 95% CIs were computed using the normal approximation, and the CIs for binomial proportions were computed by the Wilson score method [23].

Both intent-to-treat (intended participant assignment based on stepped wedge design) and subgroup (intervention received vs all controls) analyses were conducted for groupwise differences, including when comparing rates of lipid measurements in hospital and rates of discharge on high-intensity statins. Analyses evaluating discharge and follow-up outcomes excluded patients who died during hospitalization. A 2-sided *P* value of <.05 was considered statistically significant. All statistical analyses were conducted using R (version 4.1.2 software; R Core Team, R Foundation).

### Ethical Considerations

This quality improvement study was approved by the Mayo Clinic Institutional Review Board (file 21‐009289). All patients agreed to have their medical records used for research, and the institutional review board waived the need for informed consent. Subject data were deidentified in all analysis files and have been password protected within the institutional fire walls. No compensation was provided to study participants.

## Results

### Cohort Characteristics and Intervention Delivery

A total of 410 patients admitted with ACS were included in this study. Of these, 200 patients were assigned to the control group and 210 to the intervention group (Table 1). Most patients were men (285/410, 69.5%), and the overall median age at admission was 68 (IQR 60-78) years. Patients in the intervention group were slightly older than those in the control group. The most frequent ACS diagnosis was non–ST-segment elevation myocardial infarction. Unstable angina represented a greater proportion of ACS diagnoses in the intervention group than the control group. Statin use at admission was similar across this study’s groups, and almost half of patients were not taking statin medications at hospital admission. The pharmacists determined that 21/410 (5.1%) patients were not taking statin therapy due to prior intolerance, 120/410 (29.3%) patients were not taking statins because therapy had not been recommended, and 27/410 (6.6%) patients had previously declined statin therapy.

**Table 1.** Clinical characteristics of the cohort.

Characteristic			Control group (n=200)	Intervention group (n=210)	*P* value
Age (years), median (IQR)			66.5 (59‐77)	71 (61‐79.8)	.02^a^
Sex, n (%)					
Male			137 (68.5)	148 (70.5)	.66^b^
Female			63 (31.5)	62 (29.5)	
Admitting ACS^c^ diagnosis, n (%)					.003^b^
STEMI^d^			56 (28)	58 (27.6)	
NSTEMI^e^			141 (70.5)	137 (65.2)	
Unstable angina			1 (0.5)	15 (7.1)	
Other (troponin elevation)			2 (1)	0 (0)	
Admission therapy, n (%)					.51^b^
High-intensity statin^f^			56 (28)	65 (31)	
Moderate-intensity statin			52 (26)	46 (21.9)	
Low-intensity statin			8 (4)	5 (2.4)	
Nonstatin therapies			3 (1.5)	7 (3.3)	
No lipid-lowering therapy			81 (40.5)	87 (41.4)	
Inpatient LDL^g^ level (mg/dL), median (IQR)			93 (60‐127.5)	93.5 (63‐130)	.70^a^
Missing, n			45	34	
Preadmission triglyceride level (mg/dL), median (IQR; within 6 mo)			126 (90‐183.8)	149 (105.5‐215.5)	.02^a^
Missing, n			74	59	
Prior diagnosis of hyperlipidemia, n (%)			173 (88.3)	180 (85.7)	.45^b^
Missing, n			4	0	
Prior diagnosis of hypertriglyceridemia, n (%)			72 (42.9)	104 (58.4)	.004^b^
Missing, n			32	32	
Prior diagnosis of diabetes, n (%)			83 (41.7)	79 (38)	.44^b^
Missing, n			1	2	
Prior diagnosis of hypertension, n (%)			145 (72.5)	147 (70.7)	.68^b^
Missing, n			0	2	
Length of hospital stay (days), median (IQR)			3.5 (2‐10)	4 (2‐9)	.96^a^
In-hospital deaths, n (%)			3 (1.7)	5 (2.4)	.73^h^
Missing, n			19	3	
Left ventricular ejection fraction, median (IQR)			52 (38.8‐60)	55 (44‐61)	.04^a^
Missing, n			4	4	
Comorbidities, n (%)					
Prior myocardial infarction			34 (17.7)	27 (13.3)	.23^b^
Missing, n			8	7	
Prior CABG^i^			14 (7.1)	23 (11.2)	.15^b^
Missing, n			3	5	
Prior PCI^j^			63 (31.7)	54 (26.1)	.22^b^
Missing, n			1	3	
Prior diagnosis of heart failure			43 (21.5)	35 (16.7)	.22^b^
Missing, n			0	1	
Prior diagnosis of peripheral artery disease			17 (8.6)	26 (12.4)	.21^b^
Missing, n			2	1	
Prior ischemic stroke			11 (5.6)	14 (6.7)	.64^b^
Missing, n			2	0	

a Wilcoxon rank-sum test.

b Pearson chi-square test.

c ACS: acute coronary syndrome.

d STEMI: ST-segment elevation myocardial infarction.

e NSTEMI: non–ST-segment elevation myocardial infarction.

f See methods section for definitions of statin intensity.

g LDL: low-density lipoprotein.

h Fisher exact test.

i CABG: coronary artery bypass grafting.

j PCI: percutaneous coronary intervention.

Preadmission LDL test results were available for 272/410 (66.3%) participants. The median preadmission LDL was 93 (IQR 63-134) mg/dL and did not differ significantly between groups. The distribution of hyperlipidemia, hypertension, and diabetes was also similar. However, patients in the intervention group were more likely to have prior diagnosis of elevated triglycerides and slightly higher levels of preadmission triglycerides.

The median length of hospitalization was 4 (IQR 2-9) days, which was similar across this study’s groups. During hospitalization, 8 patients died, and the distribution of deaths was similar across study groups. Deaths were attributed to complications of acute myocardial infarction, including cardiogenic shock, respiratory failure from volume overload, or multisystem organ failure from persistent hypotension. The distribution was similar across this study’s groups for left ventricular ejection fraction, prior myocardial infarction, history of coronary artery bypass grafting, percutaneous coronary intervention, peripheral arterial disease, and ischemic stroke.

To assign recommendations, the pharmacists categorized patients into the following groups: taking a high-intensity statin, had a recent LDL less than 70 mg/dL; taking a high-intensity statin, had a recent LDL more than 70 mg/dL; taking a high-intensity statin, no evidence of a recent LDL measurement; taking low- to moderate-intensity statin therapy; taking lipid-lowering therapy other than a statin; and not taking lipid lowering therapy. Table 2 shows prehospital statin dosing cross-referenced with LDL values. The proportion of patients in these subgroups was not significantly different (*P*=.49).

Among the 402 patients alive at hospital discharge, the proportion of patients taking a high-intensity statin increased significantly (*P*<.001) compared with admission proportions (121/402, 30.1% to 355/402, 88.3%) including 182/205 (88.8%, 95% CI 83.4%‐92.6%) intervention participants (intent-to-treat group) and 173/197 (87.8%, 95% CI 82.2%‐91.9%) control participants (*P*=.89; Table 3). When the subgroup that received the intervention (n=100) was compared to all controls, the findings were similar.

**Table 2.** Prehospital statin therapy and low-density lipoprotein (LDL) levels of patients taking lipid-lowering therapy.^a^

Admission therapy and prehospital LDL level	Control group (n=200), n (%)	Intervention group (n=210), n (%)
HIS^b^ with LDL≤70 mg/dL	26 (13)	23 (11)
HIS with LDL>70 mg/dL	20 (10)	29 (13.8)
HIS with no recent LDL measurement	10 (5)	13 (6.2)
Low- to moderate-intensity statin	60 (30)	51 (24.3)
Nonstatin therapy	3 (1.5)	7 (3.3)
No lipid therapy	81 (40.5)	87 (41.4)

a The difference between groups was not statistically significant (*P*=.49).

b HIS: high-intensity statin.

**Table 3.** Admission and discharge medications among nondeceased patients.

Treatment	Control group (n=197), n (%)	Intervention group (n=205), n (%)
Admission therapy		
No lipid therapy	80 (40.6)	85 (41.5)
Nonstatin	3 (1.5)	6 (2.9)
Low-intensity statin	8 (4.1)	5 (2.4)
Moderate-intensity statin	50 (25.4)	44 (21.5)
High-intensity statin	56 (28.4)	65 (31.7)
Discharge therapy		
No lipid therapy	4 (2)	4 (2)
Nonstatin	4 (2)	4 (2)
Low-intensity statin	0 (0)	3 (1.5)
Moderate-intensity statin	16 (8.1)	12 (5.9)
High-intensity statin	173 (87.8)	182 (88.8)

Importantly, among patients admitted who were not receiving lipid lowering therapy, most (146/165, 88.5%) were taking a statin at discharge, and almost all patients taking a high-intensity statin at admission were taking a high-intensity statin at discharge (120/121, 99.2%). Eight patients were discharged without lipid therapy for the following reasons: 1 patient reported statin intolerance and recommendations were made to consider outpatient PCSK9 (proprotein convertase subtilisin/kexin type 9) inhibitor therapy; 1 patient had a non-ACS diagnosis at discharge, and statin therapy was appropriately withheld; 1 patient had an extremely low LDL level and preferred not to take a statin at hospital discharge; and 5 patients were discharged to hospice care and given comfort care.

The intervention was implemented for only 100/210 (47.6%) patients allocated to the intervention group, as indicated by inclusion of the templated pharmacist intervention note. Of these patients, 2 died in the hospital and 8 had recommendations coded as “other.” The pharmacist recommendations were followed (measured by the discharge medication) for 85 of the remaining 90 patients (94.4%, 95% CI 86.9%‐97.9%). See Table 4 for additional details.

**Table 4.** Pharmacist recommendations and inpatient low-density lipoprotein (LDL) measurement.

Type of delivery recommendation	Control group(n=200), n (%)	Intervention group(n=210), n (%)	*P* value^a^
Type of pharmacist EHR^b^ note			<.001
Intervention and routine notes	0 (0)	9 (4.3)	
Intervention note only	3 (1.5)	91 (43.3)	
No note or note without lipid therapy recommendation	114 (57)	62 (29.5)	
Routine notes only	83 (41.5)	48 (22.9)	
Intervention assigned and received			
Yes	N/A^c^	100 (47.6)	
Pharmacist recommendation			<.001
Continue current statin	16 (8)	19 (9)	
Continue high-intensity statin, add ezetimibe	2 (1)	5 (2.4)	
Change from admission high-intensity statin to alternative high-intensity statin	2 (1)	2 (1)	
Recommend increase in high-intensity statin dose	6 (3)	12 (5.7)	
Begin low- to moderate-intensity statin	2 (1)	1 (0.5)	
Begin high-intensity statin	25 (12.5)	61 (29)	
Begin high-intensity statin and ezetimibe	3 (1.5)	0 (0)	
Change from low- to moderate-intensity statin to a high-intensity statin	24 (12)	30 (14.3)	
No note or note without recommendation	114 (57)	62 (29.5)	
Other^d^	6 (3)	18 (8.6)	
Inpatient LDL measured	155 (77.5)	176 (83.8)	.14

a Pearson chi-square test.

b EHR: electronic health record.

c N/A: not applicable.

d Other recommendations included alternative dosing and or drug due to past statin intolerance (12 patients), recommendation to start nonstatin therapy (2 patients), transition to hospice care (1 patient), remainder were variations due to coding interpretations (9 patients).

The intent-to-treat analysis showed that 176/210 (83.8%, 95% CI 78%‐88.4%) patients in the intervention group had lipid levels measured in the hospital compared with 155/200 (77.5%, 95% CI 71%‐83%) patients in the control group (*P*=.14; Table 4). The subgroup analysis yielded a similar, nonsignificant finding (87/100, 87% vs 155/200, 77.5%; *P*=.07). Among patients who had both before and after admission LDL levels measured, their mean in-hospital LDL levels were approximately 13 mg/dL lower than they were before hospitalization (95% CI −17.9 to −7.5; *P*<.001).

### Follow-Up Period Results

Patients randomized to the intervention group were more likely to have lipid management recommendations added to the discharge summary (54/205, 26.3% vs 27/197, 13.7%; *P*=.002). Subgroup analysis showed a stronger effect, with 38/98 (38.8%) patients who received the intervention having a lipid management recommendation in their discharge summary versus 27/197 (13.7%) controls (*P*<.001). More than half (47/81, 58%) of patients with the lipid management recommendations provided in the discharge summary had LDL measured in the follow-up period compared with only 119/321 (37.1%) patients without these recommendations (*P*=.001).

Documented LDL levels within 4 weeks to 6 months of hospital discharge were available for 166/402 (41.3%) patients and included 90/205 (43.9%) of intervention patients and 76/197 (38.6%) control patients (*P*=.33; Table 5). Among the 166 patients with LDL measurements, 101 (60.8%) had a follow-up LDL of less than 70 mg/dL (median 63.5, IQR 49-79 mg/dL). The median LDL for the control group was 63 (IQR 49-79) mg/dL and for the intervention group 63.5 (IQR 49-78) mg/dL (*P*=.95). The subgroup analysis resulted in comparable findings.

**Table 5.** Low-density lipoprotein (LDL) assessment after patient discharge.^a^

LDL	Control group(n=197)	Intervention group(n=205)	*P* value
LDL measured within 4 weeks to 6 months after discharge, n (%)	76 (38.6)	90 (43.9)	.33^b^
LDL values (mg/dL), median (IQR)	63 (49‐79)	63.5 (49-78)	.95^c^

a The 8 patients who died were excluded.

b Pearson chi-square test.

c Wilcoxon rank-sum test.

## Discussion

### Principal Findings

In the intervention group of this pilot study, pharmacists provided patient-centered recommendations for guideline-directed statin therapy for patients with ACS. At hospital discharge patients in both the intervention and controls groups had very high rates of statin therapy, such that there was no significant difference for the primary outcome. However, there was significant differences in the rates of pharmacist recommendations being incorporated into the discharge summary for the intervention group and these recommendations were associated with higher rates of adjustment of statin therapy at outpatient patient follow-up. These findings demonstrate feasibility for implementation and effectiveness of the in-hospital pharmacist intervention.

The rates for patients taking a high-intensity statin were high in both the intervention and control groups. The change in therapy from admission to discharge was significant; all patients eligible and consenting to statin therapy were discharged with high-intensity therapy.

A stepped wedge cluster study design was used due to logistical constraints [22] as subjects were recruited from 6 different cardiology hospital services. These services served as natural clusters for which we delivered the intervention. Additionally, by implementing the intervention within these clusters, both the staff training and deployment of the intervention were possible. Intervention fidelity was determined by the presence of the templated pharmacist intervention note in the EHR. We found that only 100/210 (47.6%) intervention patients had this type of note documented. During this pilot, the pharmacists were not assigned to a particular service but rather served patients across multiple services. This meant pharmacists sometimes cared for both control and intervention patients in the same day, increasing the risk of low intervention fidelity (intervention patients not receiving) or intervention contamination (controls receiving the intervention). While intervention fidelity was low, there were only 3 instances of intervention templated pharmacist notes appearing in the record for a control patient demonstrating low rate of contamination.

The estimated rate of in-hospital LDL measurement was similar between this study’s groups. In both groups adherence to measuring LDL levels during hospitalization was high minimizing the opportunity to show improvement as a result of the intervention. LDL levels during hospitalization for ACS were lower than levels that were obtained within 6 months before the hospitalization for ACS event. Despite many patients having an in-patient LDL of 70 mg/dL or less during hospitalization, levels should be checked at follow-up post hospitalization as dose adjustments may be necessary. Overall, there was no difference in post hospitalization lipid measurement between the control group and the intervention group. However, intervention patients were more likely to have lipid therapy follow-up recommendations in their discharge summary, although rates were low in both groups. The subset of patients that had pharmacist recommendations for lipid testing available in the discharge summary had higher frequency of post hospital lipid measurement (*P*=.001). This suggests that communication of pharmacists’ recommendations for outpatient providers delivered via discharge summaries was beneficial, indicating that pharmacists may have an important role in bridging the gap in guideline directed care between in-hospital and outpatient care [19].

The intervention proposed herein focused on recommendations for guideline-directed optimal lipid lowering medical therapy. Diet and lifestyle modifications are also important in lipid optimization and these recommendations are routinely provided for each patient during the hospitalization by the multidisciplinary care teams. Additionally, at hospital discharge patients with ACS are routinely referred to cardiac rehabilitation programs which include comprehensive cardiovascular health assessment as well as detailed recommendations for diet and physical activity [11].

### Comparison to Previous Work

Prior studies have demonstrated a strong correlation between statin intensity and survival of patients with ACS [9]. High-intensity statins have a significant impact on survival over moderate-intensity statins regardless of patient age [9]. For this reason, our clinical practice standard is to initiate high-intensity statins on all patients hospitalized with ACS. Low use of high-intensity statins post-ACS and difficulty achieving goal LDL levels may have a negative impact on secondary prevention in patients with ACS [9].

In a prior study it was demonstrated that high-intensity statin use increased from 33.5% to 71.7% among 117,989 patients discharged from the hospital after a myocardial infarction [24]. In that same study, older age, previous statin intolerance, drug interactions, and long-term care goals were reasons that statins were not prescribed at discharge. This study showed high frequency of high-intensity statin prescription at hospital discharge, with the main reason that patients did not take statins being discharge to hospice for end-of-life care.

Previous studies demonstrated that in-hospital and follow-up lipid testing was associated with higher rates of lipid lowering therapy prescription for patients with ACS [25,26]. In this study herein, a lipid therapy recommendation in the discharge summary was associated with higher frequency of lipid testing during the follow-up period. In this study only 41% of all study patients had LDL measurements within 6 months of hospital discharge. Of these patients, 61% had an LDL less than 70 mg/dL hence nearly 40% of these patients with ACS who had follow-up lipid testing were not at goal LDL. This low frequency of follow-up lipid testing is not unique to our practice. Wang et al [27] compared data from 11,046 patients aged older than 65 years discharged from the hospital being alive from the years 2007 to 2009. In this cohort, only 44% had repeat lipid testing at 90 days and only 14% were on high-intensity statins at 1 year follow-up.

These studies highlight the need to implement interventions that improve use of lipid follow-up testing for the achievement of target LDL levels. Our proposed intervention promotes improved communication among providers including pharmacist recommendations shared across the continuum of care targeting lipid lowering therapy.

### Strengths and Limitations

The primary strength of this study is the ability to demonstrate alignment with guideline-directed high-intensity statin therapy for patients with ACS, while no overall group differences were seen this study identified an important opportunity for improved longitudinal lipid lowering therapy after hospital discharge in this high-risk population. This study suggests that a team-based approach may be successful and warrants further investigation and refinement.

This study has limitations. First, this pilot study was not randomized due to limited availability of clinical resources during this study’s period. Randomization will be used in a larger implementation trial which will be endorsed by administrative leadership for coordination and allocation of clinical resources. Second, the intervention fidelity was low, potentially diluting the treatment effect and reducing sample size for the subgroup analysis of patients who received the intervention. This reduced sample size limited statistical power for detecting group differences. There are several potential causes for the observed low intervention fidelity. A new hospital wide pharmacy initiative for documentation of pharmacist progress notes in the EHR on all patients started during this pilot. Additionally, some patients were discharged from the hospital within 24 hours after admission, which decreased the opportunity for the pharmacists to deliver the intervention. In the future, we plan to schedule activation of the intervention for a time that does not overlap with other institutional quality initiatives and improve integration of the intervention with discharge planning. Lastly, the same pharmacists were responsible for covering multiple services and sometimes cared for intervention and control patients on the same day. In the future, we plan to clearly label in the EHR which group a given patient is assigned (control vs intervention) and when possible, assign different pharmacists for control versus intervention groups. By improving intervention fidelity, statistical power for detecting group differences may also improve.

Results of this study may be generalized to other clinical settings which use team-based care in hospital practice. The institution in which this project was performed is a referral institution which may have impacted the patient population characteristics, but the care delivered was guideline-based which should be adopted in all institutions caring for patients with ACS.

### Future Directions

Shortly after this pilot study was completed, an Expert Consensus paper was published by the American College of Cardiology recommending a target LDL for high-risk (including post-ACS) patients of less than 55 mg/dL [28]. The primary driver behind this consensus document was the availability of nonstatin therapies that can further help optimize LDL levels [6]. With lower target LDL levels and the advent of nonstatin lipid lowering therapies, the proposed intervention could be adapted to lower target LDL levels and the use of both statins and nonstatin lipid lowering therapies to promote the delivery of guideline-directed care for patients with ACS.

Multidisciplinary care processes that enhance best practices for lipid management after hospital discharge of patients with ACS are needed to improve patient outcomes. A previously published study from our institution described a proactive model of care delivery assisted by clinical decision support technology to promote delivery of guideline-directed care after patients are discharged from the hospital [9]. We envision implementation of a combined process of using the pharmacist-initiated program for lipid lowering therapy in the hospital setting and a proactive outpatient model of care delivery supported by technology as described by Partogi et al [29] to promote longitudinal patient follow-up for delivery of secondary prevention guideline-directed therapy for patients with ACS.

### Conclusions

An inpatient pharmacist-initiated intervention for lipid lowering therapy for patients with ACS is feasible and effective. The main opportunity for future improvement lies in improved communication via the EHR to promote optimization of lipid management in longitudinal outpatient follow-up in this population.

## Abbreviations

ACS: acute coronary syndrome
EHR: electronic health record
LDL: low-density lipoprotein
PCSK9: proprotein convertase subtilisin/kexin type 9
REDCap: Research Electronic Data Capture
